# A GLP-1:CCK fusion peptide harnesses the synergistic effects on metabolism of CCK-1 and GLP-1 receptor agonism in mice

**DOI:** 10.1016/j.appet.2018.05.131

**Published:** 2018-08-01

**Authors:** David C. Hornigold, Emma Roth, Victor Howard, Sarah Will, Stephanie Oldham, Matthew P. Coghlan, Clemence Blouet, James L. Trevaskis

**Affiliations:** a Cardiovascular and Metabolic Diseases, MedImmune Ltd, Milstein Building, Granta Park, Cambridge, CB21 6GH, UK; b University of Cambridge, Department of Clinical Biochemistry, MRC Metabolic Diseases Unit, Addenbrooke's Hospital, Cambridge, CB2 0QQ, UK; c Cardiovascular and Metabolic Diseases, MedImmune LLC, One MedImmune Way, Gaithersburg, MD 20878, USA

## Abstract

Combination approaches for the treatment of metabolic diseases such as obesity and diabetes are becoming increasingly relevant. Co-administration of a glucagon-like peptide-1 receptor (GLP-1R) agonist with a cholecystokinin receptor-1 (CCKR1) agonist exert synergistic effects on weight loss in obese rodents. Here, we report on the effects of a novel fusion peptide (C2816) comprised of a stabilized GLP-1R agonist, AC3174, and a CCKR1-selective agonist, AC170222. C2816 was constructed such that AC3174 was linked to the N-terminus of AC170222, thus preserving the C-terminal amide of the CCK moiety. In functional *in vitro* assays C2816 retained full agonism at GLP-1R and CCKR1 at lower potency compared to parent molecules, whereas a previously reported fusion peptide in the opposite orientation, (pGlu-Gln)-CCK-8/exendin-4, exhibited no activity at either receptor. Acutely, *in vivo*, C2816 increased cFos in key central nuclei relevant to feeding behavior, and reduced food intake in wildtype (WT), but less so in GLP-1R-deficient (GLP-1RKO), mice. In sub-chronic studies in diet-induced obese (DIO) mice, C2816 exerted superior reduction in body weight compared to co-administration of AC3174 and AC170222 albeit at a higher molar dose. These data suggest that the synergistic pharmacological effects of GLP-1 and CCK pathways can be harnessed in a single therapeutic peptide.

## 1 Introduction

The prevalence of obesity and diabetes continues to rise across the globe (Heal, Gosden, & Smith, 2013). As such, therapeutic agents targeting multiple pathways are the most promising avenue to improve efficacy (Finan, Clemmensen, & Muller, 2015; Tschop et al., 2016). For example, combination approaches of small molecules (e.g., metformin, DPP4 inhibitors, SGLT2 inhibitors, etc.) have been developed for diabetes (DeFronzo et al., 2016). However, there remains a need for other combinatorial approaches that harness pharmacological synergies to maximize benefits.

CCK is released from I-cells in the upper intestine and regulates food intake via action at CCK-1 receptors (CCKR1s) (Noble et al., 1999). While a small molecule CCKR1 agonist inhibited food intake, a lower (more tolerable) dose did not significantly affect body weight in obese humans (Jordan et al., 2008). GLP-1 is released from intestinal L-cells in the more distal small intestine and colon in response to meals and regulates glucose homeostasis (Campbell & Drucker, 2013). Modified GLP-1 receptor (GLP-1R) agonists are commercially available for the treatment of diabetes and obesity. We, and others, have previously reported that CCK analogs and GLP-1R agonists interact to reduce body weight in rodents (Irwin et al., 2013; Trevaskis et al., 2015). While CCK and GLP-1 are able to exert synergistic effects on metabolism in rodents, the clinical application of administering two separate peptides is challenging.

To overcome this, Irwin et al. created a single molecular entity by linking a modified, stabilized version of CCK to a GLP-1R agonist (Irwin, Pathak, & Flatt, 2015). This molecule, (pGlu-Gln)-CCK-8/exendin-4, elicited metabolic improvements in DIO mice (Irwin et al., 2015). The linear nature of this molecule, with the C-terminus of the CCK moiety linked to the N-terminus of exenatide (abrogating the C-terminal amide group from the CCK sequence necessary for activity) appeared counter-intuitive for the retention of functional peptides of these classes. Therefore, the aim of our studies was to generate and test a fully functional dual-agonist fusion peptide utilizing the two peptide sequences that we previously confirmed interact synergistically in rodents. The fusion peptide, C2816, comprised the GLP-1R agonist peptide AC3174 linked to the CCKR1 agonist AC170222, with the C-terminus of AC170222 free to be amidated. C2816, but not (pGlu-Gln)-CCK-8/exendin-4, was determined to be a potent full agonist at both CCKR1 and GLP-1R and to reduce body weight in DIO mice.

## 2 Materials and methods

### 2.1 Peptides

AC3174 (Amylin Pharmaceuticals) is a functional analog of exenatide (Hargrove et al., 2007). AC170222 (Hpa-Nle-Gly-Trp-Lys(Tac)-Asp-NMePhe-NH_2_; CPC Scientific, Sunnyvale, CA) is a CCKR1-selective peptide (Pierson et al., 2000). C2816 is a fusion peptide of AC3174 linked to AC170222 via a mini-PEG linker (His-Gly-Glu-Gly-Thr-Phe-Thr-Ser-Asp-Leu-Ser-Lys-Gln-Met-Glu-Glu-Glu-Ala-Val-Arg-Leu-Phe-Ile-Glu-Trp-Leu-Lys-Asn-[PEG4]-Nle-Gly-Trp-Lys(Tac)-Asp-NMePhe-NH_2_); New England Peptide, Gardner, MA). (pGlu-Gln)-CCK-8/exendin-4 was synthesized by American Peptide Company (Sunnyvale, CA).

### 2.2 *In vitro* functional assays

Peptide activity at GLP-1R was determined by a cAMP accumulation assay in Chinese hamster ovary (CHO) cells stably transfected with human GLP-1 receptor (AstraZeneca), as described (Butler et al., 2015; Naylor, Rossi, & Hornigold, 2016). Eleven-point duplicate concentration response curves were generated in 3 independent experiments and data analysed as percent activation of the maximum GLP-1R ligand response. Peptide activity at CCKR1 was measured using PathHunter^®^ eXpress CCKAR CHO-K1 β-arrestin recruitment assay from DiscoverX (Fremont, CA, USA) as per manufacturer's guidelines.

### 2.3 Animal experiments

All studies were approved by the Institutional Animal Care and Use Committee at MedImmune, LLC, in accordance with Guide for the Care and Use of Laboratory Animals as adopted and promulgated by the U.S. National Institutes of Health, or in accordance with the Animals (Scientific Procedures) Act 1986 and approved by the local animal ethics committees. All mice were housed individually in standard caging at 22 °C in a 12 h light:dark cycle.

### 2.4 Induction of cFos in mice

Male 8 week-old C57/BL6J mice (Charles River Laboratories, Margate, UK) were fasted for 6 h, received an i.p. injection of saline (n = 6) or C2816 (10 nmol/kg, n = 6), anaesthetized 80 min later with pentobarbital sodium (100 mg/kg, i.p.), and received a perfusion of 50 mL of 0.01 M PBS, followed by 50 mL of 4% paraformaldehyde solution prepared in KPBS (7.0% KHPO_4_, 1.4% KH_2_PO_4_, 8.8% NaCl). Brains were post-fixed in 30% sucrose/4% paraformaldehyde at 4 °C. Brain sections were treated with H_2_O_2_, then incubated in 5% normal goat serum then a rabbit antibody raised against c-Fos (1:8000; Cat#226 003, Synaptic Systems, Goettingen, Germany) followed by standard secondary detection.

### 2.5 Acute food intake in wildtype and GLP-1R-deficient mice

Male wildtype (WT) and GLP-1R knockout mice (GLP-1RKO; 7–10 weeks of age; Jackson Laboratory, Bar Harbor, ME for MedImmune) were randomized to 4 groups (n = 10/group): WT – vehicle, WT – C2816, GLP-1RKO – vehicle, GLP-1RKO – C2816. On day −1 mice were placed in a clean cage and fasted overnight. On day 0 mice were weighed and dosed intraperitoneally (5 mL/kg) with either vehicle (PBS) or C2816 (50 nmol/kg) and pre-weighed amount of chow returned to the cage. Food was weighed periodically for 24 h, and body weight also measured at 24 h.

### 2.6 Pharmacology study in DIO mice

Obese male 10-week old C57BL6 mice (Jackson Laboratory) on high-fat diet (D12492, 60% kcal/fat, Research Diets) were single housed upon arrival and allowed to acclimate for 2 weeks. On day −3 mice were weighed and randomized to treatment groups (n = 8/group) based on body weight, with each mouse dosed once daily with two compounds or vehicle administered separately. Mice were administered vehicle/vehicle, AC3174 (7 nmol/kg)/vehicle, AC170222 (7 nmol/kg)/vehicle, AC3174 (7 nmol/kg)/AC170222 (7 nmol/kg), C2816 (50 nmol/kg)/vehicle, or (pGlu-Gln)-CCK-8/exendin-4 (50 nmol/kg). Drug was administered once daily for 10 days intraperitoneally (5 mL/kg) 2 h prior to lights off. Body weight was measured daily; on day 10 mice had non-fasted blood glucose sampled from tail nick using a glucometer (Breeze2 Ultra, Bayer), euthanized via CO_2_ inhalation and cardiac blood collected for analysis of terminal lipids and plasma enzymes (Cobas c-111, Roche Diagnostics), and insulin (MSD, Rockville, MD).

### 2.7 Kaolin intake in lean rats

Male Sprague Dawley outbred rats, aged ∼8 weeks (Envigo, Frederick, MD) were maintained on chow diet (Envigo 2018 rodent diet) and allowed to acclimate for at least 1 week. After the acclimation period, rats were given chow and kaolin diet (K50001, Research Diets, Billerica, MA) in adjacent separate compartments in a divided food hopper. Rats were randomized to treatment groups based on 24 h chow intake, 24 h kaolin intake and body weight (n = 9/group). Following an overnight fast in a clean cage, vehicle or C2816 (7 or 50 nmol/kg) was administered intra-peritoneally. Cisplatin (10 mg/kg; Sigma-Aldrich, St Louis, MO) was administered i.p. as a positive control. Chow and kaolin intake was recorded at 4 and 24 h. A final body weight was also recorded at 24 h.

### 2.8 Statistical analyses

Data were analyzed using one-way analysis of variance (ANOVA) for single-point data comparing more than two groups, or two-way ANOVA for longitudinal data. In both cases Tukey post hoc tests were performed to determine statistically significant differences between treatment groups. Significance was assumed for p < 0.05. Graphs and statistical analyses were generated using Prism 6 for Windows (Graphpad Software, San Diego, CA). For effect of C2816 on body weight in wildtype and GLP-1R knockout mice and cFos analysis Student's two-tailed *t*-test with Welch's correction was used, with a type I error probability of p < 0.05 considered significant. All data are expressed as mean ± SEM. A full statistical summary is presented in Supplementary Table 1.

## 3 Results

### 3.1 *In vitro* activity of novel GLP-1:CCK fusion peptide C2816

**Fig. 1:**
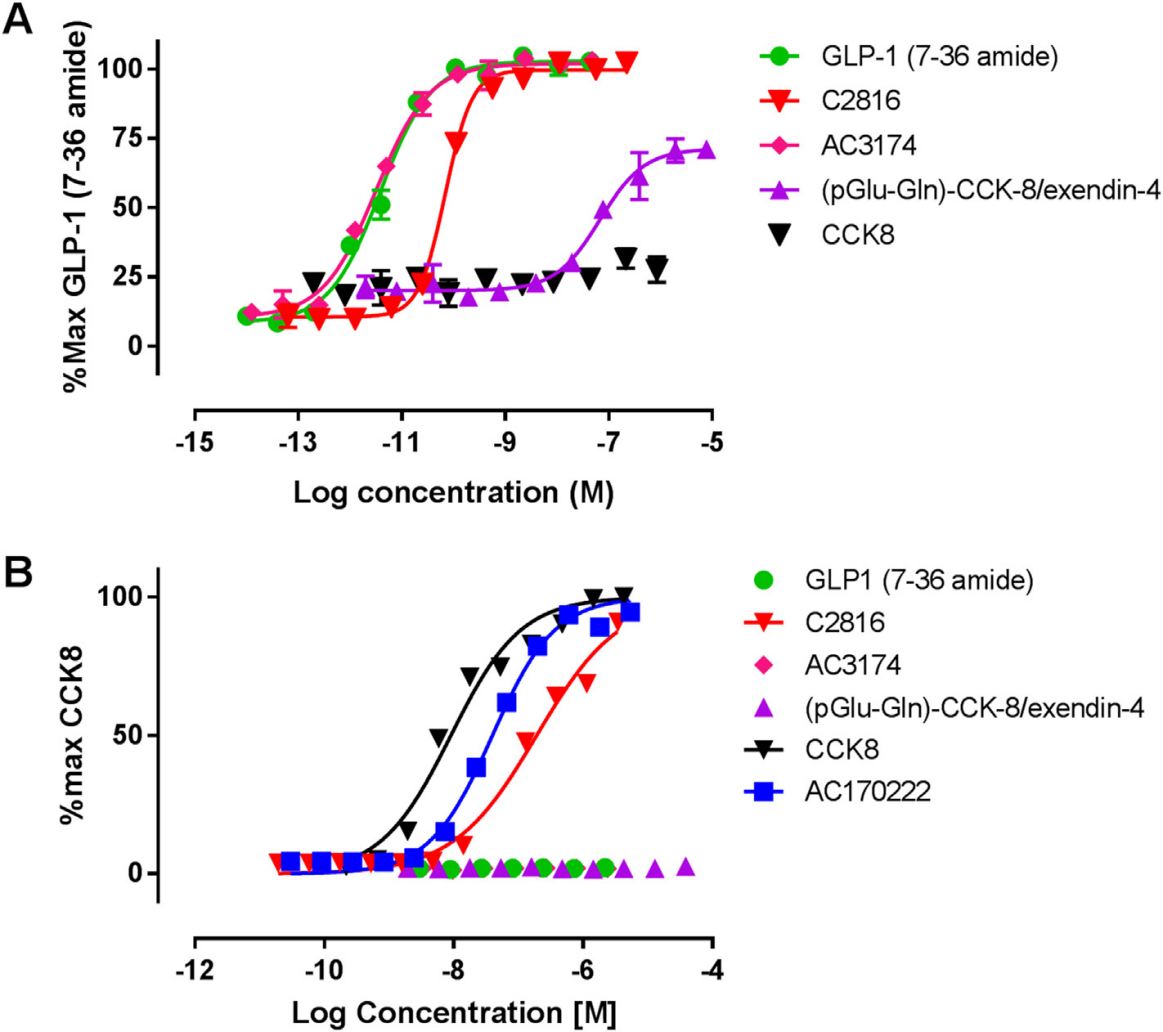
GLP-1:CCK fusion peptide C2816 is an agonist at both GLP-1 and CCK receptors. Concentration–response curves for peptide induced increase in cAMP accumulation in CHO-hGLP-1R cells **(A)** and increase in beta-arrestin recruitment in CHO- hCCK-1R cells **(B)**. Data shown are individual data points **(B)** or mean (±SD) of duplicate **(A)** fitted with 4-parameter logistic fit to determine EC_50_ and are representative of at least three independent repeats.

Potency of C2816 was assessed in cellular assays reflecting agonist activity at CCKR1 and GLP-1R relative to control peptides. Activity at GLP-1R was assessed in cAMP accumulation assay in CHO-hGLP-1R cells. C2816 acted as full agonist at hGLP-1R (Table 1, Fig. 1). C2816 was seven-fold less potent than AC3174 with EC_50_ 23.9 ± 18.0 and 3.0 ± 4.0 pM, respectively. Activity at CCKR1 was assessed in β-arrestin recruitment assay in CHO-CCK1R cells. C2816 was a full agonist with potency 16-fold weaker than AC170222 with EC_50_ of 18.1 ± 1.9, 4.6 ± 0.7 and 298.0 ± 58.7 nM for AC17022, CCK-8 and C2816 respectively (Table 1, Fig. 1). The fusion peptide (pGlu-Gln)-CCK-8/exendin-4 was inactive in CCKR1 and was a partial agonist >30,000-fold less potent than AC3174 in the GLP-1R assays with EC_50_ 99.5 ± 10.5 nM (Table 1).

**Table 1:** Determination of peptide potency (EC_50_) at GLP-1 and CCK1 receptors expressed in CHO cells. Data are geometric mean ± SEM (n). NT = not tested; NA = not active.

Peptide	Human GLP-1R cAMP assay EC_50_ (pM)	Human CCK1R β-arrestin assay EC_50_ (nM)
GLP-1 (7-36amide)	3.5 ± 0.78 (7)	NA (4)
CCK-8	NA (5)	4.6 ± 0.7 (7)
C2816	23.9 ± 18.0 (7)	298.0 ± 58.7 (5)
AC3174	3.0 ± 0.0.4 (7)	NA (4)
AC170222	NT	18.2 ± 1.9 (4)
(pGlu-Gln)-CCK-8/exendin-4	99500 ± 10500 (4)	NA (4)

### 3.2 Acute food intake in wildtype and GLP-1R-deficient (GLP-1RKO) mice

**Fig. 2:**
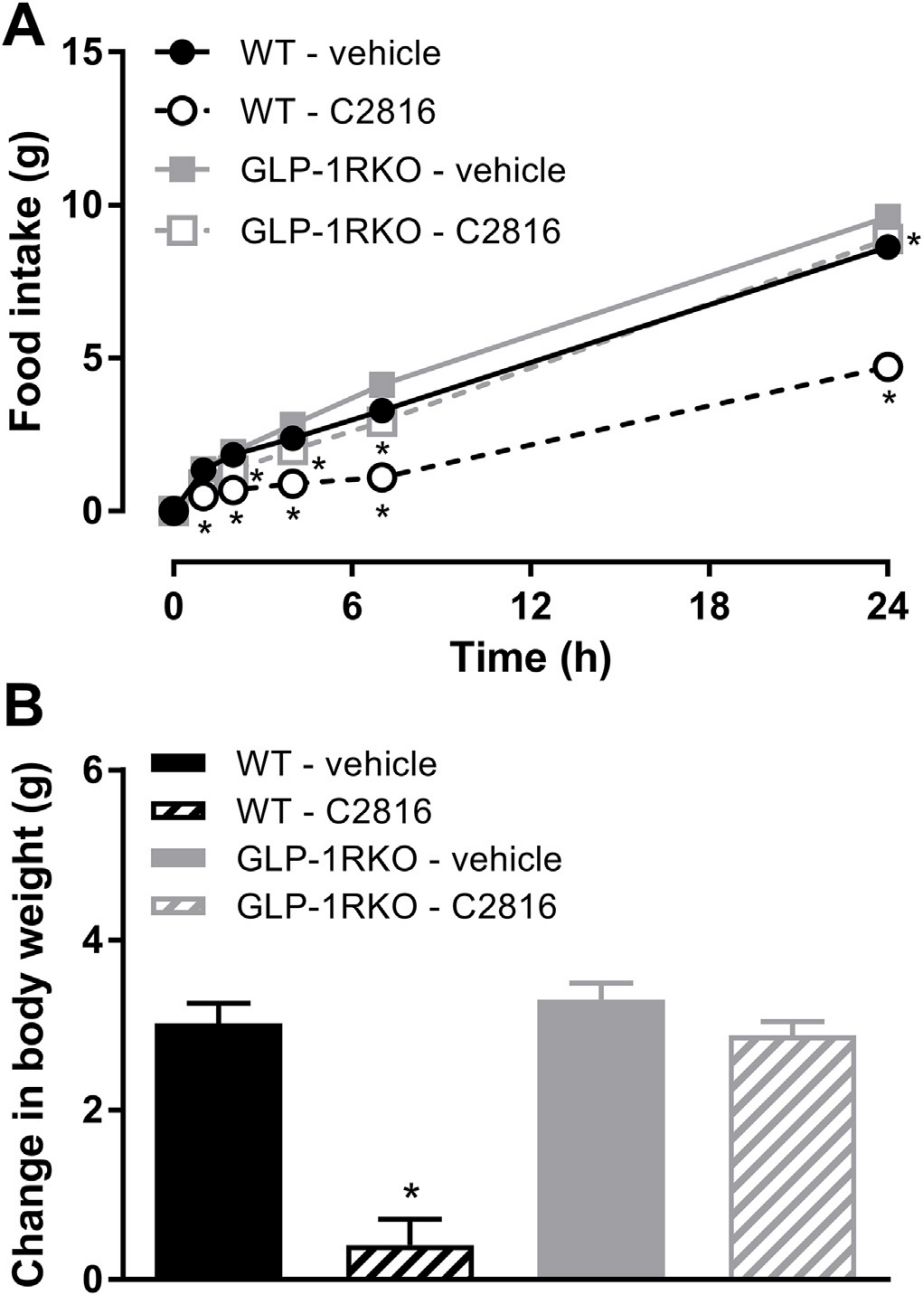
GLP-1:CCK fusion peptide C2816 reduces food intake acute in wildtype (WT) mice to a greater extent than GLP-1R-deficient (GLP-1RKO) mice, suggesting that the CCK portion of C2816 is biologically active. **(A)** 24 h food intake and **(B)** change in body weight of overnight-fasted WT or GLP-1RKO mice after a single administration of vehicle or C2816 (50 nmol/kg). *p < 0.05 vs. vehicle (within genotype).

To further confirm that the CCK portion of C2816 was biologically active we performed an acute food intake assessment in WT and GLP-1RKO mice. In overnight fasted WT mice, C2816 (10 nmol/kg) inhibited food intake relative to vehicle controls such that total 24 h food intake was suppressed by 45% (p < 0.001; Fig. 2A). Inhibition of food intake by C2816 was associated with a significant reduction in body weight (Fig. 2B). In GLP-1RKO mice treated with C2816, 24 h food intake was only suppressed by 7% relative to vehicle-treated GLP-1RKO mice (p < 0.001; Fig. 2A), and body weight was not affected (Fig. 2B).

### 3.3 C2816 activates central nuclei of the brain relevant to regulation of food intake and body weight

**Fig. 3:**
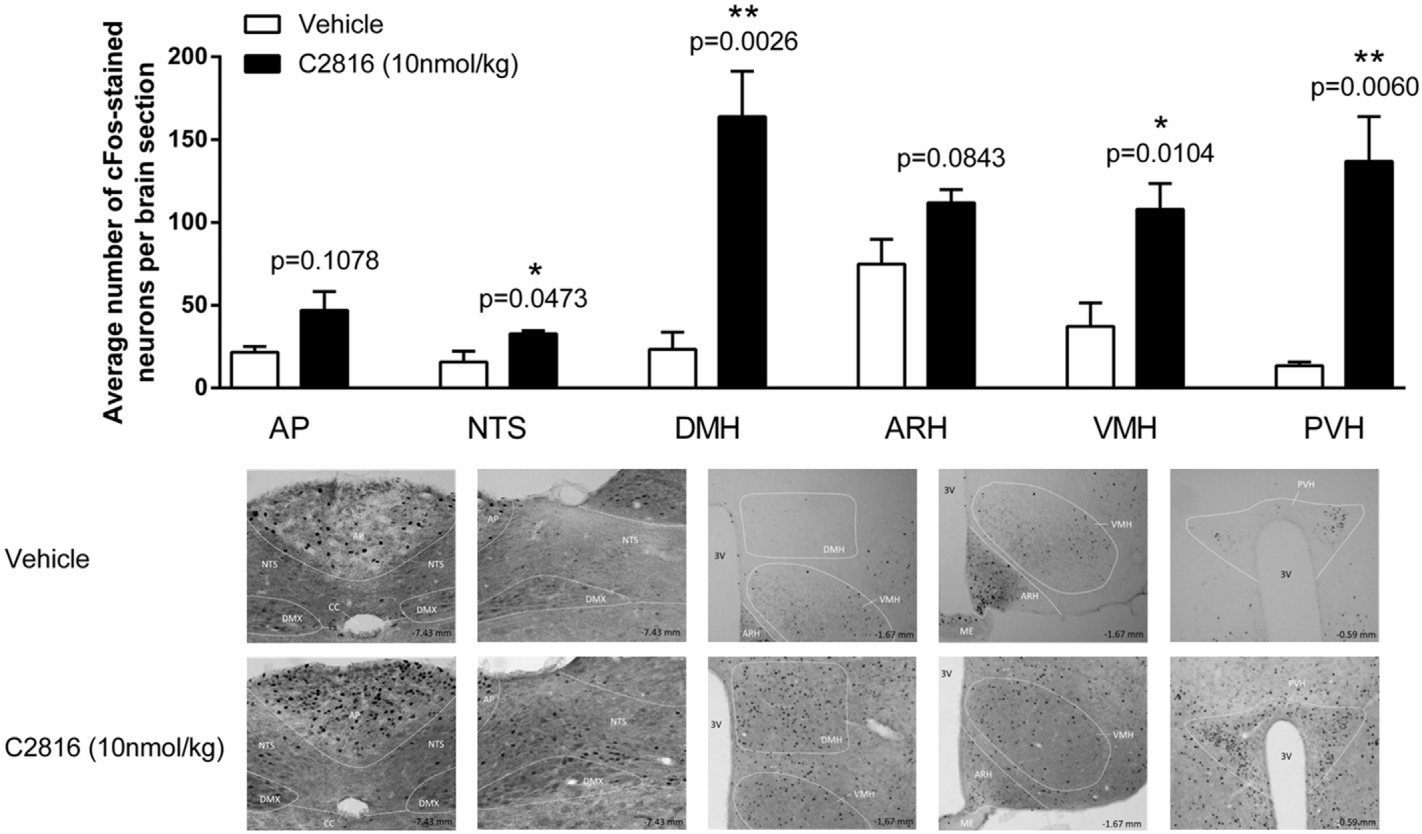
Hypothalamic and brainstem neurons in mice are acutely activated by C2816. cFos immunoreactivity in the dorsomedial, venromedial and paraventricular hypothalamic nuclei (DMH, VMH and PVH, respectively), and the nucleus tractus solitarius (NTS) and area postrema (AP) of the brainstem was quantitated 80 min following single injection of saline or C2816 (10 nmol/kg) to lean C57BL6J mice. Representative images of saline- and C2816-treated hypothalamic areas and brainstem regions are shown.

We then explored whether C2816 activated regions previously implicated in the anorectic response to GLP-1 and CCK signaling. We examined cFos immunoreactivity 80 min following a single administration of C2816 or saline in the ventromedial, dorsomedial and paraventricular nuclei of the hypothalamus (VMH, DMH, PVH) and the nucleus tract of the solitarius (NTS) and the area postrema (AP) in the caudomedial brainstem (Blevins, Stanley, & Reidelberger, 2000; Rinaman, 2010; Secher et al., 2014). At the 10 nmol/kg dose tested C2816 significantly increased cFos signal in the VMH, DMH and PVH relative to saline controls (Fig. 3). In the brainstem, C2816 also increased cFos in the NTS (Fig. 3) but not the AP (Fig. 3).

### 3.4 Sustained administration of C2816 reduces body weight in DIO mice

**Fig. 4:**
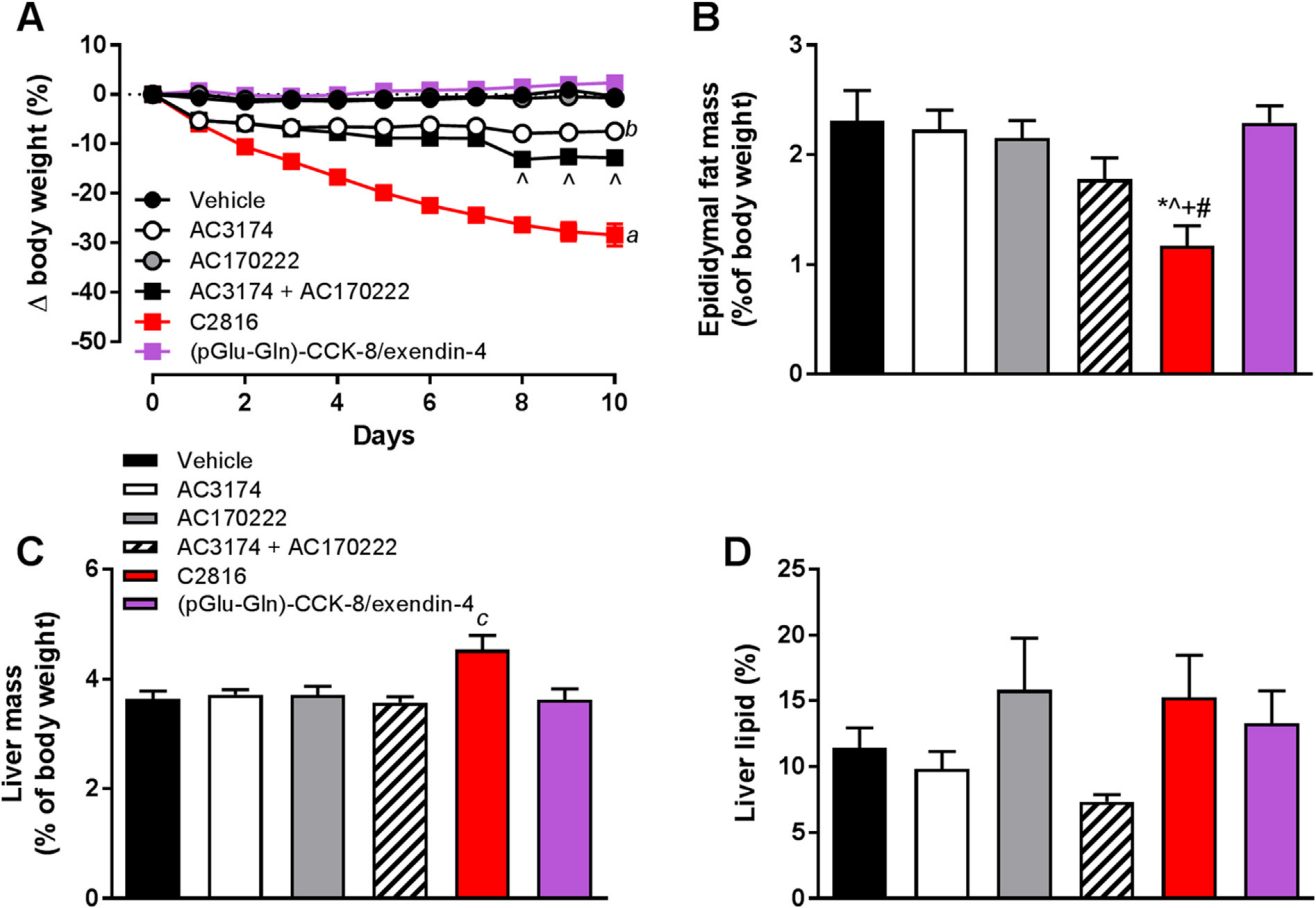
Sustained administration of C2816 induces body weight loss and metabolic improvements in DIO C57BL6 mice, superior to co-infusion of CCKR1 agonist peptide (AC170222) and GLP-1 analog (AC3174) parent molecules. *p < 0.05 vs. vehicle, ˆp < 0.05 vs. AC3174, +p < 0.05 vs. AC170222, #p < 0.05 vs. (pGlu-Gln)-CCK-8/exendin-4, ^*a*^p<0.05 for C2816 group vs. all other groups at all study days, ^*b*^p < 0.05 for AC3174 vs. vehicle, AC170222 and (pGlu-Gln)-CCK-8/exendin-4 groups at all study days, ^*c*^p < 0.05 vs. all other groups.

Administration of AC170222 to DIO mice did not affect body weight, whereas AC3174 induced significant (−7.4%) body weight loss (Fig. 4A). Co-administration of AC3174 and AC170222 further enhanced weight loss (−12.8%) relative to AC3174 alone (Fig. 4A). C2816 profoundly reduced body weight by −28.4% vs. vehicle and was significantly lower than all other groups (Fig. 4A). In agreement with *in vitro* data, (pGlu-Gln)-CCK-8/exendin-4 was ineffective. C2816 reduced epididymal fat and liver weight (Fig. 4B-C), however liver lipid content was not altered (Fig. 4D). Blood glucose, plasma triglycerides and cholesterol were reduced in C2816 group vs. all other groups and insulin tended to be lower (Table 2). Plasma ALT was elevated in C2816 vs. AC3174, and AST and plasma lipase was higher in C2816 group vs. all other groups (Table 2). Amylase was higher in C2816 mice relative to AC3174 and (pGlu-Gln)-CCK-8/exendin-4 groups (Table 2).

**Table 2:** Terminal blood glucose, and plasma concentrations of liver enzymes, lipids and exocrine pancreatic enzymes in diet-induced obese mice following 10 days of treatment with vehicle, AC3174, AC17022, combination of AC3174 + AC170222, fusion peptide C2816, or (pGlu-Gln)-CCK-8/exendin-4. *p < 0.05 vs. vehicle, ˆp < 0.05 vs. AC3174, +p < 0.05 vs. AC170222, #p < 0.05 vs. AC3174 + AC170222; †p < 0.05 vs. (pGlu-Gln)-CCK-8/exendin-4.

	Vehicle	AC3174	AC170222	AC3174 + AC170222	C2816	(pGlu-Gln)-CCK-8/exendin-4
Glucose (mg/dL)	298 ± 15	266 ± 15	280 ± 11	260 ± 12	183 ± 9*ˆ+#†	314 ± 11#
Insulin (ng/mL)	14.3 ± 5.2	6.4 ± 0.7	11.3 ± 4.1	5.0 ± 0.5	2.1 ± 0.8	13.8 ± 4.8
ALT (U/L)	56 ± 7	57 ± 15	86 ± 32	53 ± 6	132 ± 27#	56 ± 11
AST (U/L)	79 ± 11	71 ± 11	95 ± 27	67 ± 6	204 ± 52*ˆ+#†	81 ± 10
Cholesterol (mg/dL)	213 ± 6	219 ± 13	192 ± 28	164 ± 15	73 ± 17*ˆ+#†	192 ± 19
Triglycerides (mg/dL)	112 ± 10	108 ± 7	114 ± 6	94 ± 8	73 ± 7*ˆ+†	118 ± 11
Amylase (U/L)	2667 ± 94	2795 ± 83	2759 ± 75	2516 ± 131	2275 ± 171ˆ†	2858 ± 115
Lipase (U/L)	25 ± 1	50 ± 13	77 ± 49	118 ± 77	373 ± 103*ˆ+#†	46 ± 17

### 3.5 C2816 does not induce pica in lean rats

**Fig. 5:**
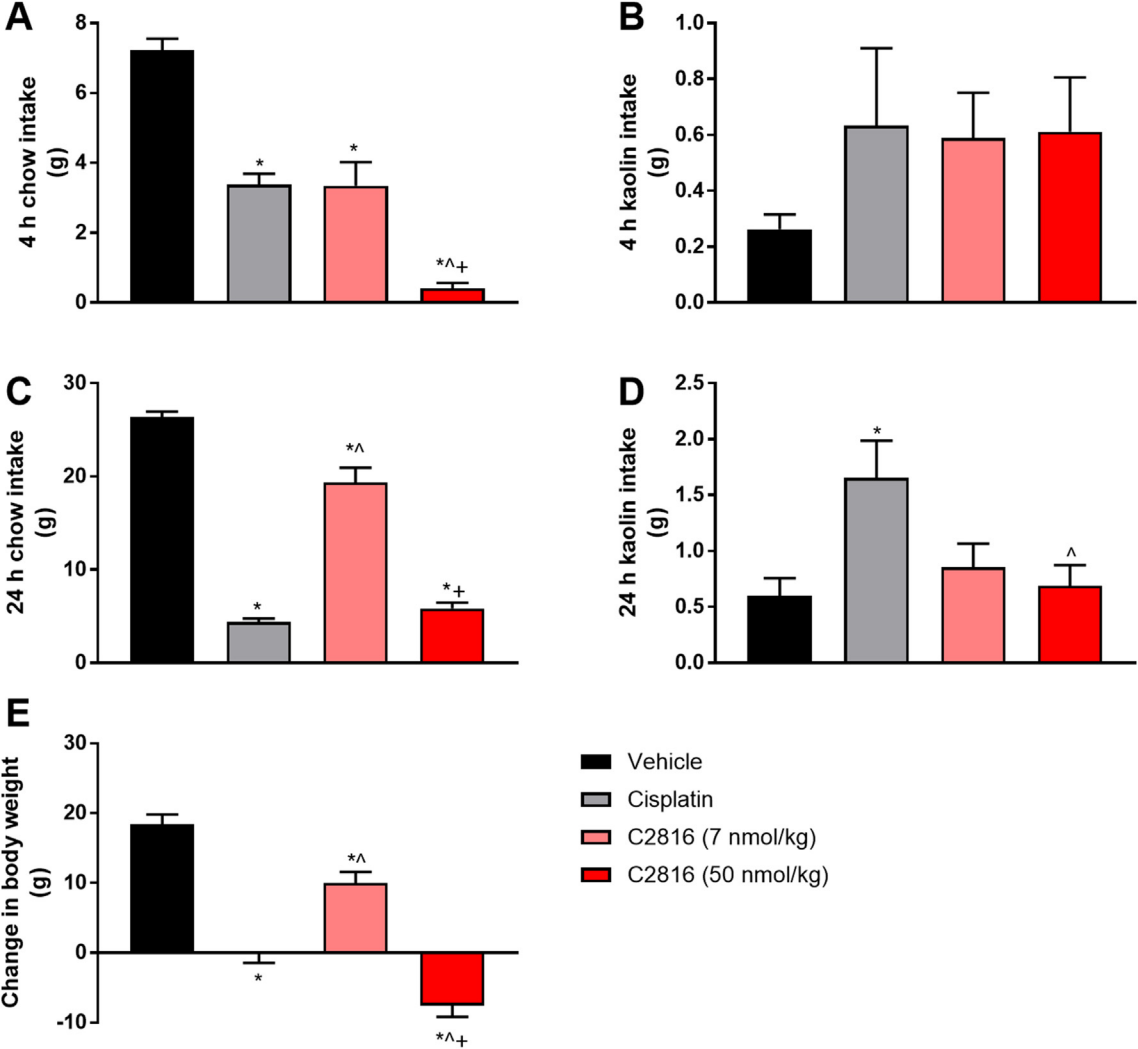
Acute administration of C2816 does not induce pica behavior in lean rats. Intake of chow at 4 h and 24 h **(A, C)**, and the non-nutritive clay kaolin **(B, D)** in overnight fasted Sprague Dawley rats administered vehicle, cisplatin (10 mg/kg) or C2816 (7 or 50 nmol/kg). Change in body weight over the 24 h observation period was also recorded **(E)**. *p < 0.05 vs. vehicle, ˆp < 0.05 vs. cisplatin, +p < 0.05 vs. C2816 (7 nmol/kg).

We assessed the potential for C2816 to induce nausea/malaise in rodents via determination of kaolin intake following acute administration after an overnight fast in lean rats. Food intake was significantly reduced by C2816 in a dose-dependent manner after single injection vs. vehicle controls at 4 h and 24 h post-dose (Fig. 5A, C). Inhibition of food intake corresponded with a reduction in overall body weight (Fig. 5E). Neither dose of C2816 was associated with increased kaolin intake (Fig. 5B, D), while administration of cisplatin which similarly reduced food intake as high dose C2816 did increase 24 h kaolin consumption (Fig. 5D).

## 4 Discussion

To harness two distinct pathways for the treatment of metabolic disorders we generated a fusion peptide linking two peptides previously validated as exerting synergistic benefits on body weight (Trevaskis et al., 2015). The concept of a CCK:GLP-1 fusion peptide has been previously explored (Irwin et al., 2015). Our molecule is conceptually similar but in the opposite orientation (GLP-1:CCK), in order to retain the free C-terminal amide group on the CCK moiety. Further, modifications of the N-terminus of GLP-1/exenatide typically impair function (Gallwitz et al., 1994). Thus, a GLP-1:CCK fusion peptide, which we termed C2816, was generated. *In vitro* characterization revealed that C2816 retained full agonist activity at both human CCKR1 and GLP-1R, with slightly reduced potency. Biological activity of the CCK moiety of C2816 was demonstrated by acute feeding studies in GLP-1RKO mice, where a small but statistically significant effect of C2816 was observed, suggesting some biological effect of the CCK portion of C2816. The clear reduction in efficacy of C2816 in GLP-1RKO mice suggests a heavy reliance on GLP-1R however caution should be taken. The ability of C2816 to inhibit food intake in WT mice reflects not just engagement with each receptor specifically, but also the synergistic component. That is, at certain dose levels where CCK and GLP-1 exert synergistic effects, studies in single receptor knockout mice may not fully capture the contribution of each receptor to the overall sum effect. To date, such detailed food intake studies have not been performed. Furthermore, to gain a fuller understanding of the contribution of each part of C2816 similar studies in mice lacking CCKR1 would be necessary. Without an equivalent food intake assessment in CCKR1-deficient mice to determine the sole contribution of GLP-1R (without synergy) the question as to whether C2816 is able to harness synergy or merely capture partial activity of the parent molecules remains unanswered.

In a sustained dosing study in DIO mice C2816 exerted greater weight loss relative to co-administration of the parent molecules, however some experimental conditions need to be considered. Firstly, the doses of the parent molecules used was less than C2816 on a molar ratio. We anchored our dose strategy around a maximally effective dose (∼30 μg/kg or 7 nmol/kg) of AC3174 (Hargrove et al., 2007). The dose of C2816 was adjusted for the reduced potency at GLP-1R (7-fold), leading to a final dose selection of 50 nmol/kg for C2816. Thus, if all the biological effects of C2816 were mediated via GLP-1R then the efficacy would be similar between C2816 and the AC3174 treatment arm. Secondly, the pharmacokinetic profiles and/or tissue distribution may be different between C2816 and parent molecules. Finally, full activity profiles at mouse receptors remains unknown and may diverge from human receptor assays. With these caveats, C2816 exerted substantially greater weight loss compared to co-administration of parent molecules supporting the notion that activation of CCKR1 is a major contributor to the overall efficacy of C2816. The nature of C2816-mediated weight loss remains to be fully characterized, however it is likely that the weight loss is, at least in part, mediated via central effects given the cFos signal generated by C2816 in brain regions relevant to energy balance regulation. These effects also appear to be independent of malaise-inducing or emetic responses as C2816 failed to induce kaolin intake in lean rats at doses that significantly reduced food intake and body weight. The lack of pica behavior following C2816 administration is in line with previous observations showing similar lack of kaolin intake in rats treated with AC3174 in combination with several doses of CCK8 (Trevaskis et al., 2015). Overall, the data show that the weight-reducing effects of C2816 may be mediated centrally and independently of malaise/nausea. Effects on other aspects of energy balance such as energy expenditure or physical activity remain to be determined.

The mechanism/s of GLP-1 and CCK that can be harnessed pharmacologically to induce synergistic effects on metabolism is/are unknown. The key question that remains is: what is the minimal level of CCKR1 agonism that can be safely used in conjunction with a GLP-1 that induces synergy, and could such a potency ratio be harnessed in a single peptide? We demonstrated that full agonism of each receptor is possible with a single peptide and it is likely that other molecules with exquisite potency ratios can be generated. Further, the orientation of such peptides is also important. It is clear that C2816, with the GLP-1-to-CCK orientation, retained largely full biological effects whereas the (pGlu-Gln)-CCK-8/exendin-4 molecule with the opposite CCK-to-GLP-1 orientation did not, exhibiting only partial GLP-1R agonism. As such, the (pGlu-Gln)-CCK8/exendin-4 molecule, in our hands, did not elicit any biological activity. Without more definitive analysis of compound exposure or *in vivo* target engagement we cannot explain the discrepancy between our observations and the reported efficacy of (pGlu-Gln)-CCK8/exendin-4. The optimal orientation (and subsequent implications on specific modulation of N- and C-terminal modifications to the peptides and resultant disruption in potency and biological activity as mentioned previously) and potency ratios of GLP-1:CCK fusion peptides beyond that exemplified by C2816 will be required to explore the question of the minimally effective contribution of CCKR1 agonism to enhance GLP-1R pharmacotherapy in future studies.

In conclusion we generated a dual agonist fusion peptide, C2816, harnessing both GLP-1R and CCKR1 pathways to induce significant reductions in weight in obese mice. This molecule represents a preliminary foray into GLP-1:CCK fusion peptides, highlighting the potential utility of this approach as an example of combination therapy to treat metabolic disease.
