# Supplementary material for: A GLP-1:CCK fusion peptide harnesses the synergistic effects on metabolism of CCK-1 and GLP-1 receptor agonism in mice
Source: Appetite. 2018 Aug 1;127:334–40. doi: 10.1016/j.appet.2018.05.131 (PMC6026274; doi:10.1016/j.appet.2018.05.131)
Supplement: Multimedia component 1 [file mmc1.docx]

**Supplementary Table 1.** Full statistical summary for experiments presented.

| **Figure** | **Statistical analyses** | **F (DFn, DFd)** | **t, df** | **P-value** |
| --- | --- | --- | --- | --- |
| Figure 1A  *Food intake* | Two-way repeated measures ANOVA, Tukey’s post hoc test | Interaction: F (15, 180) = 71.33 |  | p < 0.0001 |
| Figure 1B  *Total body weight change (wildtype)* | Unpaired t-test  (two-tailed) |  | t=6.856, df=18 | p < 0.0001 |
| Figure 1B  *Total body weight change (GLP-1RKO)* | Unpaired t-test  (two-tailed) |  | t=1.687, df=18 | p = 0.1088 |
| Figure 2  *Area postrema (AP)* | Unpaired t-test  (two-tailed) |  | t=2.13 df=3.602 | p = 0.1078 |
| Figure 2  *Nucleus of solitary tract (NTS)* | Unpaired t-test  (two-tailed) |  | t=2.499 df=5.896 | p = 0.0473 |
| Figure 2  *Dorsomedial hypothalamus (DMH)* | Unpaired t-test  (two-tailed) |  | t=4.791 df=6.311 | p = 0.0026 |
| Figure 2  *Arcuate nucleus (ARH)* | Unpaired t-test  (two-tailed) |  | t=2.179 df=4.727 | p = 0.0843 |
| Figure 2  *Ventromedial hypothalamus (VMH)* | Unpaired t-test  (two-tailed) |  | t=3.353 df=7.791 | p = 0.0104 |
| Figure 2  *Paraventricular hypothalamus (PVH)* | Unpaired t-test  (two-tailed) |  | t=4.547 df=5.06 | p = 0.0060 |
| Figure 4A  *Change in body weight* | Two-way repeated measures ANOVA, Tukey’s post hoc test | Interaction: F (40, 350) = 47.64 |  | p < 0.0001 |
| Figure 4B  *Fat mass* | One-way ANOVA, Tukey’s post hoc | F (5, 42) = 5.171 |  | p = 0.0009 |
| Figure 4C  *Liver weight* | One-way ANOVA, Tukey’s post hoc | F (5, 42) = 4.597 |  | p = 0.0020 |
| Figure 4D  *Liver lipid* | One-way ANOVA, Tukey’s post hoc | F (5, 42) = 1.797 |  | p = 0.1344 |
| Figure 5A  *4 h chow intake* | One-way ANOVA, Tukey’s post hoc | F (3, 31) = 42.46 |  | p < 0.0001 |
| Figure 5B  *4 h kaolin intake* | One-way ANOVA, Tukey’s post hoc | F (3, 31) = 0.7587 |  | p = 0.5259 |
| Figure 5C  *24 h chow intake* | One-way ANOVA, Tukey’s post hoc | F (3, 31) = 124.7 |  | p < 0.0001 |
| Figure 5D  *24 h kaolin intake* | One-way ANOVA, Tukey’s post hoc | F (3, 31) = 4.25 |  | p = 0.0126 |
| Figure 5E  *Change in body weight* | One-way ANOVA, Tukey’s post hoc | F (3, 31) = 55.41 |  | p < 0.0001 |
